# EEG and gut microbiota response patterns in high-altitude indigenous populations

**DOI:** 10.1128/msystems.01692-25

**Published:** 2026-03-04

**Authors:** Ke Bai, Ting Ge, Chen-Xi Wang, Yi-Yi Dou, Ji-Xuan Zhang, Peng Li, Xiu-Long Feng, Yu Han, Sha-Sha Zhao, Kui-Ming Su, Yu-Xuan Shang, Xing Yu, Si-Rui Li, Dan Su, Jia-Jie Song, Xu Qin, Jie Yu, Chang-Bin Yang, Jun-Peng Zhang, Wen Wang

**Affiliations:** 1 Department of Radiology, Tangdu Hospital, Fourth Military Medical University 12644, Xi'an, Shaanxi, People’s Republic of China; 2 Functional and Molecular Imaging Key Lab of Shaanxi Province, Xi'an, Shaanxi, People’s Republic of China; 3 7T MRI Precision Neurology Platform of Shaanxi Province, Xi'an, Shaanxi, People’s Republic of China; 4 Institute of Basic Medicine, Fourth Military Medical University, Xi'an, Shaanxi, People’s Republic of China; 5 Military Medical Innovation Center, Fourth Military Medical University, Xi'an, Shaanxi, People’s Republic of China; 6 Department of Automation, College of Electrical Engineering, Sichuan University 12530, Chengdu, Sichuan, People’s Republic of China; Istanbul Medipol University School of Medicine, Istanbul, Türkiye

**Keywords:** high altitude, electroencephalography (EEG), gut microbiota, brain/gut interaction

## Abstract

Indigenous high-altitude populations maintain relatively normal brain function despite chronic hypoxia, yet the underlying neurophysiological mechanisms and the potential role of gut-brain interaction remain unclear. This study combined 16S rRNA gut microbiota profiling in 211 high-altitude indigenous populations at 2, 3, and 4 km altitudes with resting-state and task-based electroencephalography recordings in 135 of them. Residents at 4 km showed enhanced delta (1–4 Hz) power across most brain regions along with increased frontal-occipital functional connectivity (FC) during resting state. During a cognitive oddball task, the 4 km group exhibited elevated P3 amplitude in response to oddball stimuli, together with larger parietal delta power. In parallel, the 4 km group displayed higher species richness and an elevated abundance of short-chain fatty acid-producing genera such as *Roseburia*, *Blautia*, and *Coprococcus*. Furthermore, the abundance of *Blautia* was positively associated with resting-state FC, a relationship that may further influence anxiety and sleep quality. Our findings demonstrate a coordinated gut-brain interaction adaptation to high altitude, highlighting the homeostatic role of microbial pathways.

**IMPORTANCE:**

Indigenous high-altitude populations maintain normal cognitive function under chronic hypoxia, a process potentially involving the gut microbiota. Our study added evidence that the neural activity patterns and gut microbiota structure may work in coordination to assist the host in adapting to extreme environments.

## INTRODUCTION

High-altitude environments (>2,500 m) impose chronic hypobaric hypoxia, aridity, extreme temperature fluctuations, and intense ultraviolet radiation (1), posing severe physiological challenges to human beings. The human brain, with its high oxygen demand and exquisite sensitivity to hypoxia, is particularly susceptible to altitude (2). In lowlanders, acute high-altitude exposure can induce memory impairment (3), attention deficits (4), emotional dysregulation (5), and sleep disturbance (6). Indigenous high-altitude populations, especially Tibetans, exhibit remarkable preservation of neurological and cognitive function despite lifelong hypoxia. This resilience is attributed to unique genetic (e.g., *EPAS1* variants) and physiological adaptations acquired over generations (7–9). Furthermore, genetic differences contribute to distinct adaptation mechanisms among ethnic groups residing in high-altitude regions (10). However, the neurophysiological mechanisms that maintain brain health under chronic hypoxia remain to be fully elucidated.

Neuroimaging has provided some evidence by directly observing the unique adaptive brain changes among high-altitude residents. Electroencephalography (EEG) studies have reported a nonlinear relationship between high-altitude exposure and neurophysiological changes (11): initially increased cognitive resource consumption during early processing stages, followed by gradual recovery and potential enhancement through physiological adaptation. Migrant-based studies have also reported increased resting-state functional connectivity (FC) in the occipital lobe (12) and more efficient brain network reorganization during executive control tasks (13), potentially optimizing perceptual and motor coordination. However, indigenous residents have experienced hypoxia since gestation and throughout critical neurodevelopmental periods and possess distinct genetic adaptations. Consequently, migrant-based findings cannot be readily extrapolated to them (14). Direct evidence of lifelong neural adaptations in indigenous high-altitude residents therefore remains scarce.

The gut microbiota, a symbiotic community of microorganisms in the gastrointestinal tract, functions as a dynamic interface between environmental stressors and host physiological processes. Emerging evidence suggests that gut microbiota is a key modulator under high-altitude environment stress (15, 16). High-altitude residents exhibit distinct microbial profiles, such as more abundant phylum *Firmicutes* (17, 18), many of which are major producers of short-chain fatty acids (SCFA). Within the brain, they regulate neuroinflammation and help maintain barrier integrity (19), and further influence synaptic plasticity and neurotransmitter synthesis (20). These combined effects may contribute to high-altitude acclimatization. Although both brain functional alterations and gut microbiota shifts have been widely reported in high-altitude adaptation, their potential interplay among indigenous populations remains unclear.

The present study tested the hypothesis that lifelong residence at high-altitude environments elicits coordinated gut microbiota and cerebral activity adaptations. To control for potential genetic confounding and isolate the effects of high-altitude exposure, we focused on a Han Chinese population. Resting-state and task-based EEG were acquired from indigenous populations living at 2 km, 3 km, and 4 km, respectively. Meanwhile, the 16S rRNA sequencing of their gut microbiota was performed. Resting-state analyses focused on power spectral density (PSD) and FC, whereas task-state analyses examined event-related potential (ERP) and time-frequency dynamics during a visual oddball task. Correlation analyses were performed to examine the associations between microbial features and neural indices. This study aimed to investigate the link between high-altitude-induced gut microbiota shifts and neurophysiological adaptation and to elucidate the potential role of gut-brain interaction.

## MATERIALS AND METHODS

### Participants

This cross-sectional study enrolled 211 healthy, native-born Han Chinese adults with lifelong continuous residency at the respective altitudes. Participants were categorized by residential altitude into three groups: 2 km group (*n* = 70, mean age 34.4 ± 10.1 years, 43 females and 27 males), 3 km group (*n* = 72, mean age 36.7 ± 10.2 years, 33 females and 39 males), and 4 km group (*n* = 69, mean age 34.9 ± 12.1 years, 31 females and 38 males). All participants were normal or corrected-to-normal vision and were right-handed. They had no history of neurological or psychiatric disorders, severe cardiovascular diseases, including major myocardial infarction, arrhythmias, chronic pulmonary diseases, diabetes, hypertension, or other systemic conditions, and no history of smoking or alcohol abuse.

### Neuropsychological assessment

Neuropsychological assessments were conducted under the direct supervision of two trained mental health professionals. The assessment battery included the self-rated anxiety scale (SAS) and the self-rated depression scale (SDS) for mood evaluation, the Pittsburgh sleep quality index (PSQI) and the Epworth sleepiness scale (ESS) for sleep quality assessment. The California verbal learning test (CVLT) was performed to evaluate verbal learning and memory through five trials with 16 semantically categorized words, short-delay (5-min) and long-delay (20-min) recall following an interference list instruction, and a final recognition trial.

### Gut microbiota analysis

#### Sample collection and DNA extraction

Fresh fecal samples were self-collected immediately after neuropsychological testing using sterile collection kits. Total genomic DNA was extracted using the OMEGA Soil DNA Kit (M5635-02; Omega Bio-Tek, GA, USA) according to the manufacturer’s protocol. The extracted DNA was stored at −20°C before analysis. The quantity and quality of extracted DNA were assessed with a NanoDrop NC2000 spectrophotometer (Thermo Fisher Scientific, MA, USA) and agarose gel electrophoresis.

#### 16S rRNA gene sequencing

The V3–V4 region of the 16S rRNA gene was amplified using the universal primers 338F (5′-ACTCCTACGGGAGGCAGCA-3′) and 806R (5′-GGACTACHVGGGTWTCTAAT-3′), with 7 bp barcodes added for multiplex sequencing on an Illumina NovaSeq 6000 platform under 2 × 250 bp paired-end configuration. Following sequencing, data were processed in QIIME 2 (21) through demultiplexing and primer trimming with cutadapt (22). Sequences were then quality-filtered, denoised, merged, and chimera-filtered using DADA 2 (23) to generate amplicon sequence variants (ASVs). ASVs were aligned with mafft (24), a phylogenetic tree was constructed using FastTree 2 (25), and taxonomic classification was assigned via a naive-Bayes classifier trained on the Greengenes2 reference database (26).

#### Bioinformatics and statistical analysis

Sequence data analyses were mainly performed using QIIME2 and R packages. Alpha-diversity indices (Chao1, observed ASVs, Shannon, and Simpson) were calculated, and beta-diversity was assessed using Bray-Curtis distances. Between-group differences in the relative abundance of microbial taxa were tested with the Kruskal-Wallis test followed by Bonferroni post-hoc tests. Venn diagrams were constructed to display the number of ASVs that were shared among or unique to different sample groups. Differentially abundant microbial taxa were identified using linear discriminant analysis (LDA) effect size (LEfSe), with LDA score (log_10_) > 2 as the threshold for biomarker detection. A random forest analysis was conducted to identify discriminant microbial features for altitude groups, with feature importance assessed and recursively refined using recursive feature elimination. The functional potential of the microbial communities was predicted using Phylogenetic Investigation of Communities by PICRUSt2 (27), and mapped to the Kyoto Encyclopedia of Genes and Genomes (KEGG) pathways for enrichment analysis.

### EEG signal recording

EEG data were acquired from 135 out of 211 participants using a 32-channel Neuroscan system with eego software in an electrically shielded chamber. Ag/AgCl electrodes were positioned according to the international 10–20 system, referenced online to bilateral mastoids (M1/M2), with ground at GND and impedance kept below 5 kΩ. To reduce the recording and analysis burden, the EEG signals were sampled at 500 Hz. All participants were abstained from coffee, alcohol, and psychoactive medications for 24 h preceding the experiment and maintained regular sleep schedules. Resting-state EEG was recorded for 5 min, during which participants maintained eyes-closed wakefulness and minimized movement to reduce artifacts. Subsequently, the visual oddball task was conducted, during which stimulus presentation and data recording were performed using E-Prime 2.0. Random digits appeared centrally, with a standard stimulus of “8” (80% probability) and a target of “2” (20% probability). Each trial commenced with an 800 ms central fixation cross “+,” followed by the digit (50 ms) and a blank screen with a randomized duration between 1,000 and 1,200 ms. Participants pressed the left mouse button for target “2” only. Each participant completed 200 trials.

### Resting-state EEG analysis

#### Signal processing

Raw EEG data were preprocessed using EEGLAB in MATLAB 2021b (MathWorks, Natick, MA, USA). Continuous recordings underwent sequential filtering: 1 Hz high-pass, 40 Hz low-pass, and 48–52 Hz notch filtering. All epochs were visually inspected to exclude those containing prominent artifacts. Independent component analysis removed artifact components including blinks, eye movements, muscular activity, and cardiac interference. Epochs exceeding ±100 μV amplitude were rejected. Finally, the data were re-referenced to the whole-brain average and segmented into 2-s epochs for each participant.

#### PSD analysis

PSD was estimated from 1 to 30 Hz using Welch’s method (28) with a 1-s sliding Hanning window, 50% overlap, and 1-Hz frequency resolution. The mean PSD was subsequently converted to decibel units (dB/Hz) via logarithmic transformation (10·log_10_). Frequency bands of interest were defined as delta (1–4 Hz), theta (4–7 Hz), and alpha (7–13 Hz), respectively. For each frequency band, intergroup differences were assessed across frontal (F3, Fz, and F4), central (C3, Cz, and C4), parietal (P3, Pz, and P4), and occipital (O1, Oz, and O2) electrode regions.

#### FC analysis

FC was assessed using the weighted phase lag index (WPLI) to quantify phase synchronization strength between neural oscillations in EEG signals (29). WPLI weights the cross-spectrum according to the magnitude of the imaginary component, ranging from 0 (no synchronization) to 1 (perfect synchronization). For each participant, the 30 × 30 connectivity matrices were derived by computing WPLI at every analyzed frequency and time point. Representative values were subsequently extracted by averaging WPLI within the delta, theta, and alpha bands, respectively.


$${\text{WPLI}}_{xy}=\frac{n^{-1}\sum_{t=1}^{n}|\text{lmag}\left(s_{xyt}\right)|sgn(\text{imag}\left(s_{xyt}\right))}{n^{-1}\sum_{t=1}^{n}|\text{imag}\left(S_{xyt}\right)|}$$

### Task-state EGG analysis

#### Signal processing

Continuous recordings underwent sequential filtering: 0.1 Hz high-pass, 40 Hz low-pass, and 48–52 Hz notch filtering. Data were segmented into −600 to 1,000 ms epochs relative to stimulus onset for target and standard stimuli and were baseline-corrected using the −600 to 0 ms interval. After excluding trials with incorrect responses, all epochs were visually inspected to remove those containing prominent artifacts. Independent component analysis removed artifact components including blinks, eye movements, muscular activity, and cardiac interference. Epochs exceeding ±100 μV amplitude were rejected. Finally, data were re-referenced to the averaged bilateral mastoid electrodes (M1 and M2). For ERP analysis, epochs from −200 to 1,000 ms relative to stimulus onset were extracted and baseline-corrected from −200 to 0 ms. For time-frequency analysis, epochs from −600 to 1,000 ms relative to stimulus onset were extracted with baseline correction from −400 to −200 ms.

#### ERP analysis

Based on prior research (30–32) and observed waveform characteristics in the current study, component-specific time windows and electrode sites were defined as follows: 320–480 ms for the P3 component and 140–180 ms for the N1 component. Amplitudes were then averaged across the parietal electrodes (P3, Pz, and P4). For each participant, ERP waveforms were derived by averaging all trials separately for oddball and standard stimuli. Component latency was subsequently calculated for each identified peak.

#### Time-frequency analysis

Time-frequency data were analyzed within the 1–30 Hz frequency range, with power changes calculated using a 400-ms Hanning window at 1-Hz frequency step and 2-ms time step. Post-stimulus power was converted to the decibel (dB) scale using the transformation (10 × log_10_ [power(*t*)/power(baseline)]). Finally, for each participant, time windows of interest were selected as 350–500 ms for the delta and theta bands and 150–250 ms for the alpha band. Power values were then averaged across the parietal electrodes (P3, Pz, and P4).

### Statistical analyses

Statistical analyses were performed using SPSS software (Version 28.0; IBM Corp., Armonk, NY, USA). Normality and homogeneity of variance were assessed for all data sets. Demographic and neuropsychological variables were analyzed using the Kruskal-Wallis test. Categorical variables were analyzed using the chi-square test. Spectral power differences across frontal (F3, Fz, and F4), central (C3, Cz, and C4), parietal (P3, Pz, and P4), and occipital (O1, Oz, and O2) regions were examined using one-way ANOVA. ERP and time-frequency data were analyzed using 2 (condition: oddball vs standard) × 3 (altitude: 2, 3, and 4 km) ANOVA. Spearman’s correlation analysis was used to assess the associations between EEG metrics and microbiota abundance, with anxiety, depression, sleep quality, and the CVLT recognition scores included as covariates in regression models. All tests were two-tailed with the significance level set at α = 0.05. False discovery rate (FDR) correction was applied for FC analyses, and Bonferroni correction was used for other *post hoc* multiple comparisons. A comprehensive statistical summary is provided in Table S1.

## RESULTS

### Demographic and neuropsychological information

The demographic characteristics of the 211 participants are summarized in Table 1. No significant differences were observed for age, sex, and education among the three groups. Indigenous population at 4 km reported higher anxiety (SAS; vs 2 km: *P* = 0.008; vs 3 km: *P* = 0.010) and depression scores (SDS; vs 3 km: *P* = 0.003), and poorer sleep quality (PSQI; vs 2 km: *P* = 0.012). The 2 km group showed a higher CVLT recognition number than the 3 km group (*P* < 0.001; Fig. S1). From the 135 EEG recordings, 5 participants were excluded from resting-state and 14 from task-state analyses due to excessive artifacts or poor task performance. The final EEG samples were balanced across groups with respect to age, sex, and education. In summary, indigenous population at the highest altitude was associated with worse mood, sleep quality, and verbal memory.

**TABLE 1:** Group demographics^*a*^

Variable	2 km (*n* = 70)	3 km (*n* = 72)	4 km (*n* = 69)	*K*/*χ*^2^	*P* value
Age, years	36.00 (12.00)	37.50 (16.00)	35.00 (20.00)	1.811	0.404
Sex, M/F	27/43	39/33	38/31	4.826	0.090
Education, years	9.00 (9.00)	7.50 (6.00)	12.00 (10.00)	5.249	0.072

^*a*^ Sex was analyzed using the chi-square test, while age and education were analyzed using the Kruskal-Wallis test. Data are presented as median (interquartile range).

### PSD results

**Fig 1:**
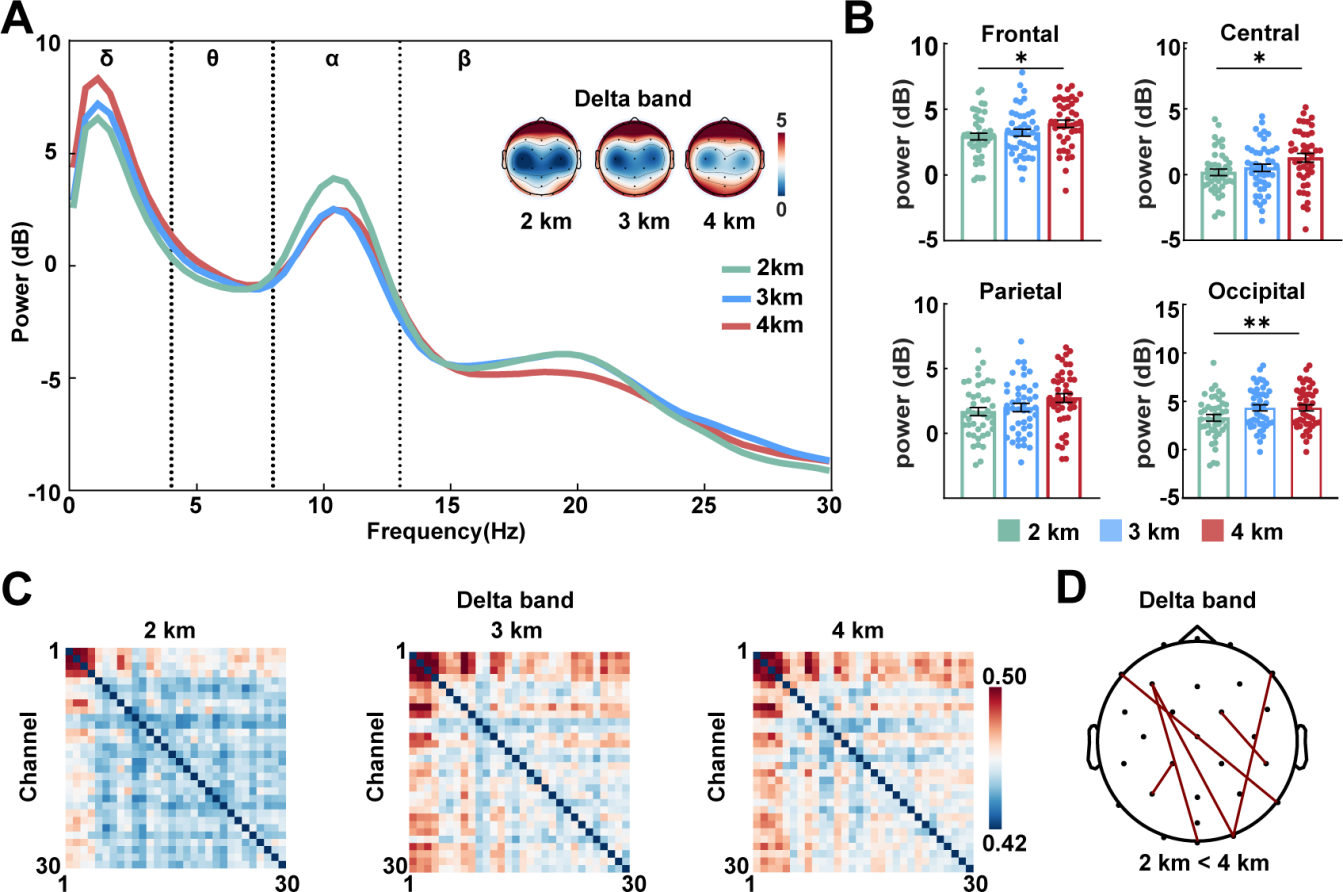
Resting-state EEG characteristics. (**A**) Grand average power spectra across all scalp electrodes with topographic maps of the delta band. (**B**) Delta power (1–4 Hz) across selected electrode clusters covering major cortical regions: frontal (F3, Fz, and F4), central (C3, Cz, and C4), parietal (P3, Pz, and P4), and occipital (O1, Oz, and O2) electrodes. Data are presented as mean ± SEM. Statistical analyses were using one-way ANOVA followed by Bonferroni *post hoc* tests for each electrode region. (**C**) Grand average FC matrix in the delta band. (**D**) FC with significant differences between the 2 and 4 km groups in the delta band. Pairwise group comparisons were performed using independent samples *t*-tests, with FDR correction. *P < 0.05 and **P < 0.01. EEG, electroencephalography; FC, functional connectivity.

Grand average oscillatory power spectra across delta, theta, alpha, and beta are presented in Fig. 1A. Significant between-group differences in delta-band power were identified across frontal (*F* = 4.326, *P* = 0.015), central (*F* = 3.718, *P* = 0.027), and occipital clusters (*F* = 6.208, *P* = 0.003). *Post hoc* comparisons confirmed significantly larger delta power in the 4 km group compared to the 2 km group at frontal (*P* = 0.016), central (*P* = 0.026), and occipital clusters (*P* = 0.002; Fig. 1B). No significant differences were observed in theta or alpha bands. Delta-band FC was enhanced in the 4 km group relative to the 2 km group, particularly between frontal and occipital regions (Fig. 1C and D). No other significant intergroup difference was observed (*P* > 0.05). These results indicate that high-altitude exposure was linked to enhanced low-frequency oscillatory activity and FC in the resting brain.

### ERP results

**Fig 2:**
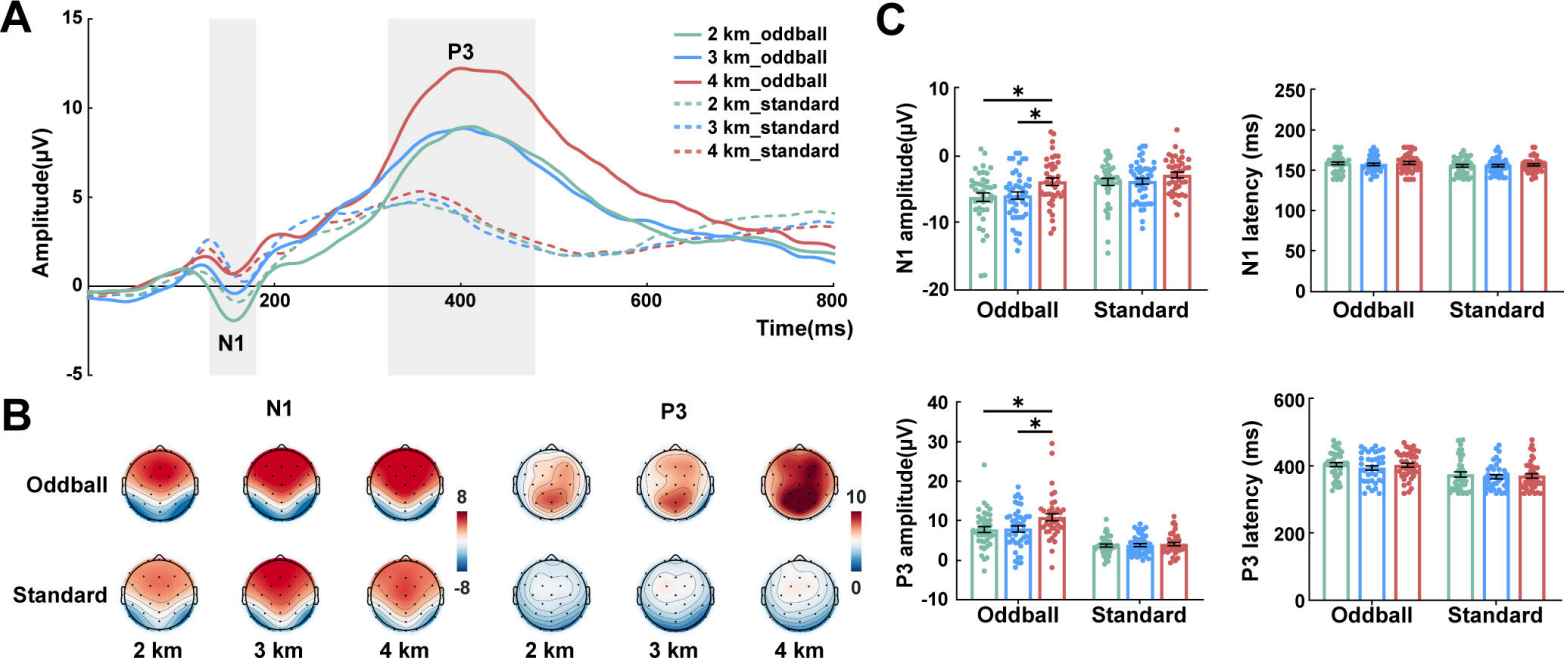
The ERP characteristics of the oddball task. (**A**) Grand average ERP waveforms elicited by standard and oddball stimuli at parietal electrodes (P3, PZ, and P4). (**B**) Topographic maps of the N1 component (140–180 ms) and the P3 component (320–480 ms). (**C**) Amplitude and peak latency of the N1 and P3 components. Data are presented as mean ± SEM. Statistical analyses were using mixed-design ANOVA followed by Bonferroni *post hoc* tests for each ERP component. *P < 0.05. ERP, event-related potential.

The waveform and topographic maps of P3 and N1 are shown in Fig. 2A and B. Behavioral performance (accuracy and reaction time) did not differ across altitude groups or stimulus conditions (Table S2). For the N1 component, analysis confirmed a significant group main effect (*F* (2, 118) = 3.271, *P* = 0.041, *ηp*² = 0.053) and a significant interaction between stimulus and group (*F* (2, 118) = 6.631, *P* = 0.002, *ηp*² = 0.101). *Post hoc* tests demonstrated higher N1 amplitudes to oddball stimuli in the 4 km group than in the 2 km group (*P* = 0.020) and the 3 km group (*P* = 0.031), with no significant differences observed for standard stimuli. For the P3 component, a significant main effect of group was observed (*F* (2, 118) = 2.922, *P* = 0.058, *ηp*² = 0.047), along with a statistically significant stimulus and group interaction (*F* (2, 118) = 4.650, *P* = 0.011, *ηp*² = 0.073). The 4 km group exhibited larger P3 amplitudes than the 2 km group (*P* = 0.021) and the 3 km group (*P* = 0.035). In contrast, no significant differences were found for standard stimuli. Peak latency did not differ significantly across groups (Fig. 2C). Taken together, these ERP alterations suggested that high altitude affected distinct stages of attentional processing, manifesting as enhanced N1 and P3 of oddball stimuli.

### Time-frequency results

**Fig 3:**
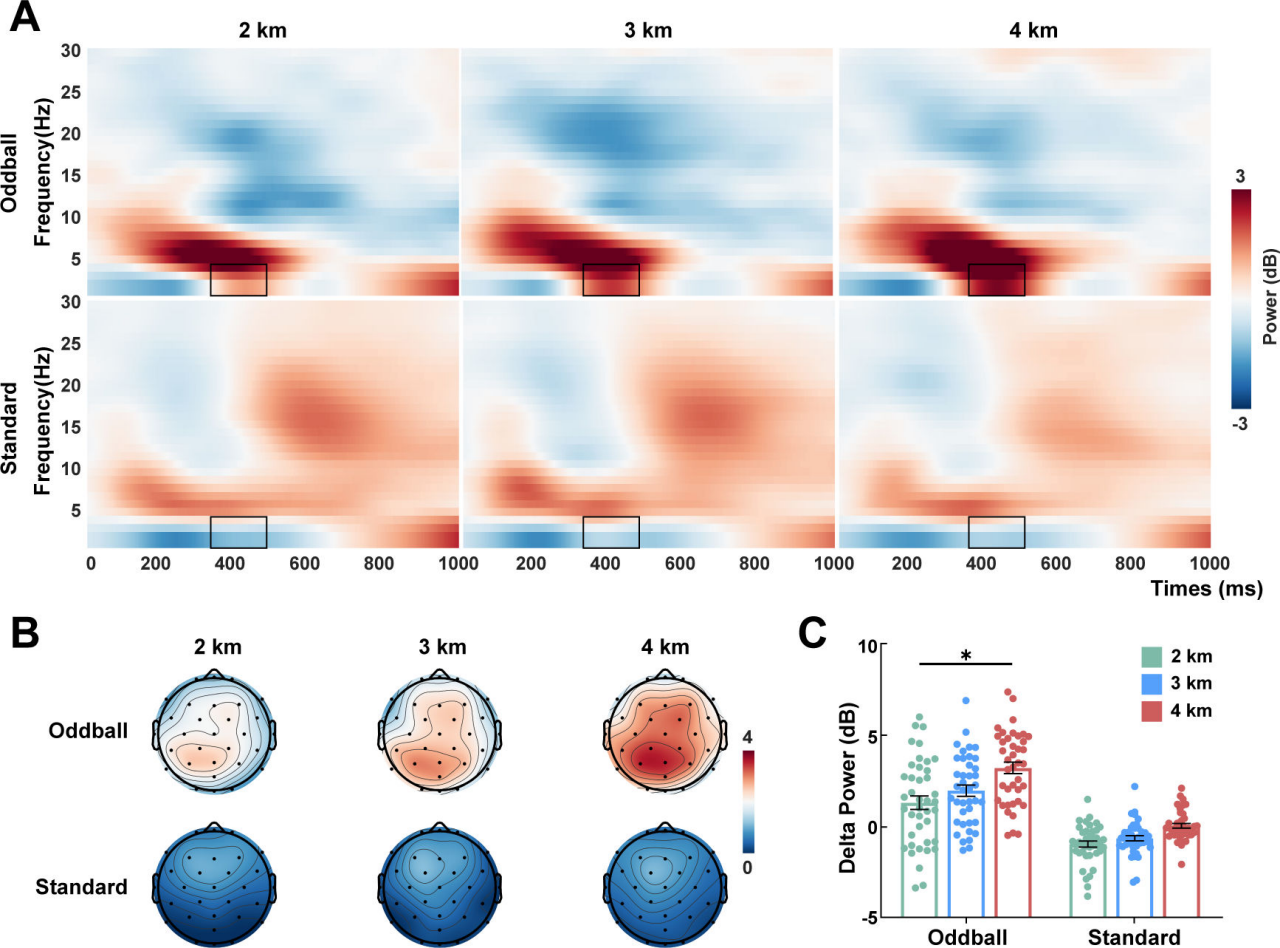
The time-frequency characteristics of the oddball task. (**A**) Grand average time-frequency distributions for standard and oddball stimuli at parietal electrodes (P3, Pz, and P4). (**B**) Topographic maps of the delta band (350–500 ms) (**C**) Mean power of the delta band. Data are presented as mean ± SEM. Statistical analyses were using mixed-design ANOVA followed by Bonferroni *post hoc* tests. *P < 0.05.

Grand average time-frequency computed across all electrodes for three groups are displayed in Fig. 3A. Significant delta band (350–500 ms) activity at parietal electrodes (P3, Pz, and P4) was observed (Fig. 3B). Statistical analysis indicated significant main effect of group on delta power (*F* (2, 118) = 3.697, *P* = 0.028, *ηp*² = 0.059), and a considerable stimulus and group interaction (*F* (2, 118) = 2.829, *P* = 0.063, *ηp*² = 0.046). *Post hoc* tests demonstrated that during oddball stimuli, the 4 km group exhibited larger delta power compared to the 2 km group (*P* = 0.020). In contrast, no significant between-group differences emerged for standard stimuli (Fig. 3C). Theta and alpha bands activity showed neither significant (Fig. S2). This pattern indicates the association between high altitude and increased delta-frequency neural activity during cognitive engagement.

### Composition of differential microbiota

**Fig 4:**
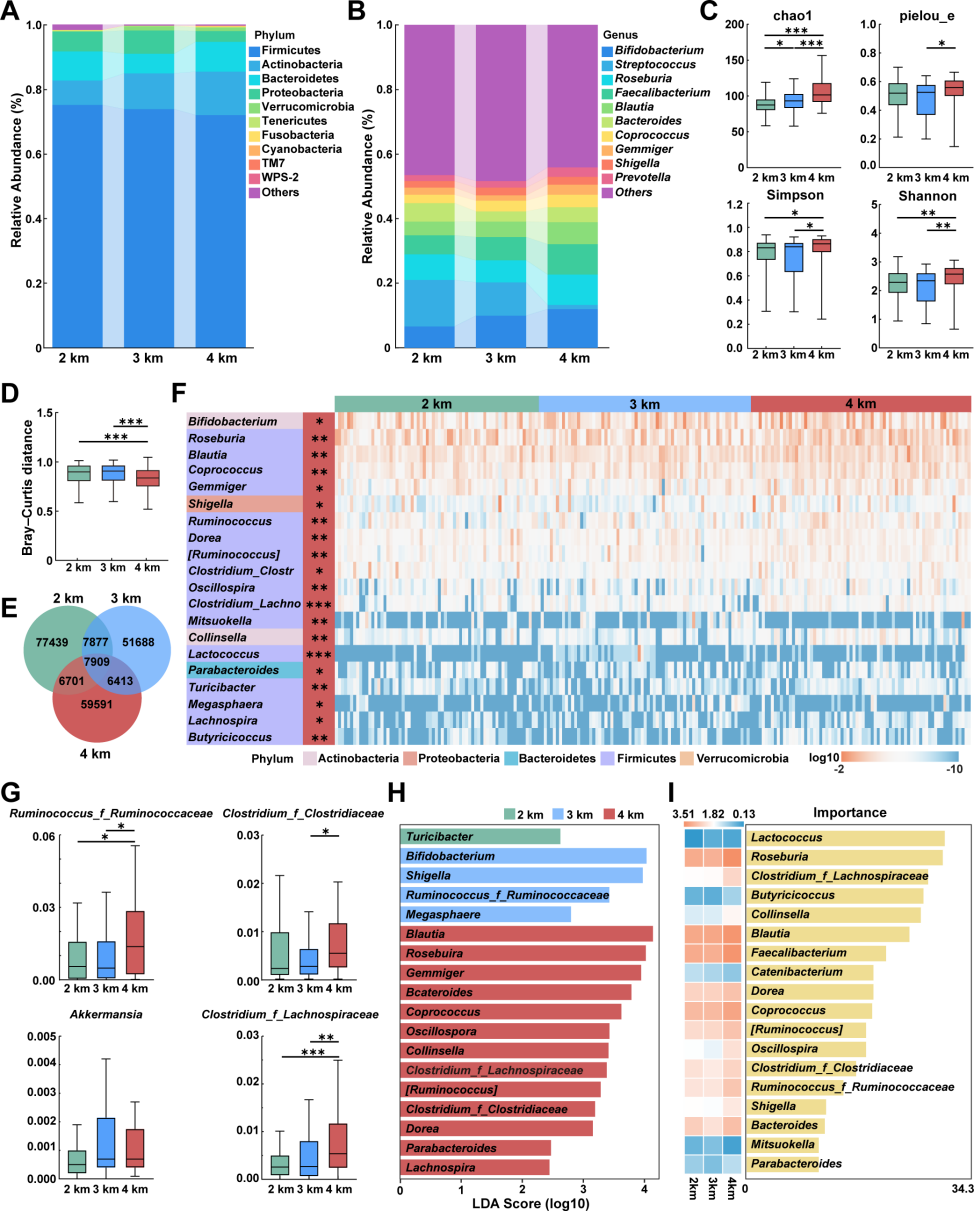
Diversity of the gut microbiome characteristics. (**A**) The alluvial plot of relative abundance of the 10 most abundant phyla. (**B**) The alluvial plot of the relative abundance of the 10 most abundant genera. (**C**) Alpha diversity. Statistical analyses were using the Kruskal-Wallis test with Bonferroni *post hoc* tests. (**D**) Bray-Curtis distance. Data are presented as median and IQR. Statistical analyses were using the Kruskal-Wallis test with Bonferroni *post hoc* tests. (**E**) A Venn diagram delineates unique and shared ASVs stratified by group. (**F**) Relative abundance heatmap of the top 20 genera among those with significant inter-group differences. The left panel lists taxonomic assignments (genera and phyla), the middle column indicates the group of significant enrichment for each indicator, and the right panels display the log_10_ relative abundance per sample. The *P* values shown here were not adjusted for multiple comparisons. (**G**) Comparison of the relative abundance of several genera. Data are presented as median and IQR. Statistical analyses were using the Kruskal-Wallis test with Bonferroni *post hoc* tests. (**H**) Linear discriminant analysis (LDA) effect size (LEfSe) analysis identified differentially abundant microbiota, applying thresholds of LDA scores (log_10_) > 2.0 and *P* < 0.05. (**I**) Classification analyses based on random forest models. *P < 0.05, **P < 0.01, and ***P < 0.001.

16S rRNA sequencing of fecal samples yielded 15,421,758 high-quality reads (mean: 73,088.9 per sample, range: 43,235–124,023). Following rarefaction to an even depth of 29,678 reads, 200,143 ASVs were identified. Community composition analysis at phylum and genus levels revealed *Firmicutes* as the dominant phylum, followed by *Actinobacteria*, *Bacteroidetes*, and *Proteobacteria* (Fig. 4A). At the genus level, *Bifidobacterium*, *Streptococcus*, and *Roseburia* were dominant (Fig. 4B). Compared to the 2 km group, the 4 km group exhibited significantly greater community richness, as measured by the Chao1 index (*P* < 0.001), Simpson index (*P* = 0.021), and Shannon index (*P* = 0.008; Fig. 4C). Bray-Curtis distances confirmed that the gut microbiota composition differed significantly across the altitude groups (*P* < 0.001; Fig. 4D). The Venn diagram showed 7,909 shared ASVs across all samples, with 77,439, 51,688, and 59,591 unique ASVs in the 2, 3, and 4 km groups, respectively (Fig. 4E).

Genus-level comparative analysis revealed that the 4 km group exhibited elevated abundances of *Roseburia* (*P* = 0.026), *Blautia* (*P* = 0.002), and *Coprococcus* (*P* = 0.004; Fig. 4F) compared to the 2 km group. Notably, most differential genera clustered in the 4 km group, predominantly *Firmicutes* members, including *Ruminococcus* (family *Ruminococcaceae*) and *Clostridium* from both the *Lachnospiraceae* and *Clostridiaceae* families (Fig. 4G). LEfSe analysis identified a distinct microbial taxon pattern among altitudes (Fig. 4H), with 1 bacterial taxon enriched in the 2 km group, 4 in the 3 km group, and 13 in the 4 km group. Among these, *Blautia*, *Roseburia*, and *Gemmiger* were dominant in the 4 km group (LDA > 2.0). Random forest modeling identified *Lactococcus*, *Roseburia*, and *Clostridium* (family *Lachnospiraceae*) as the top genus ranked by feature importance (Fig. 4I). In summary, high-altitude indigenous populations were associated with a distinct gut microbial signature marked by increased diversity and the enrichment of SCFA-producing genera.

### Functional prediction

**Fig 5:**
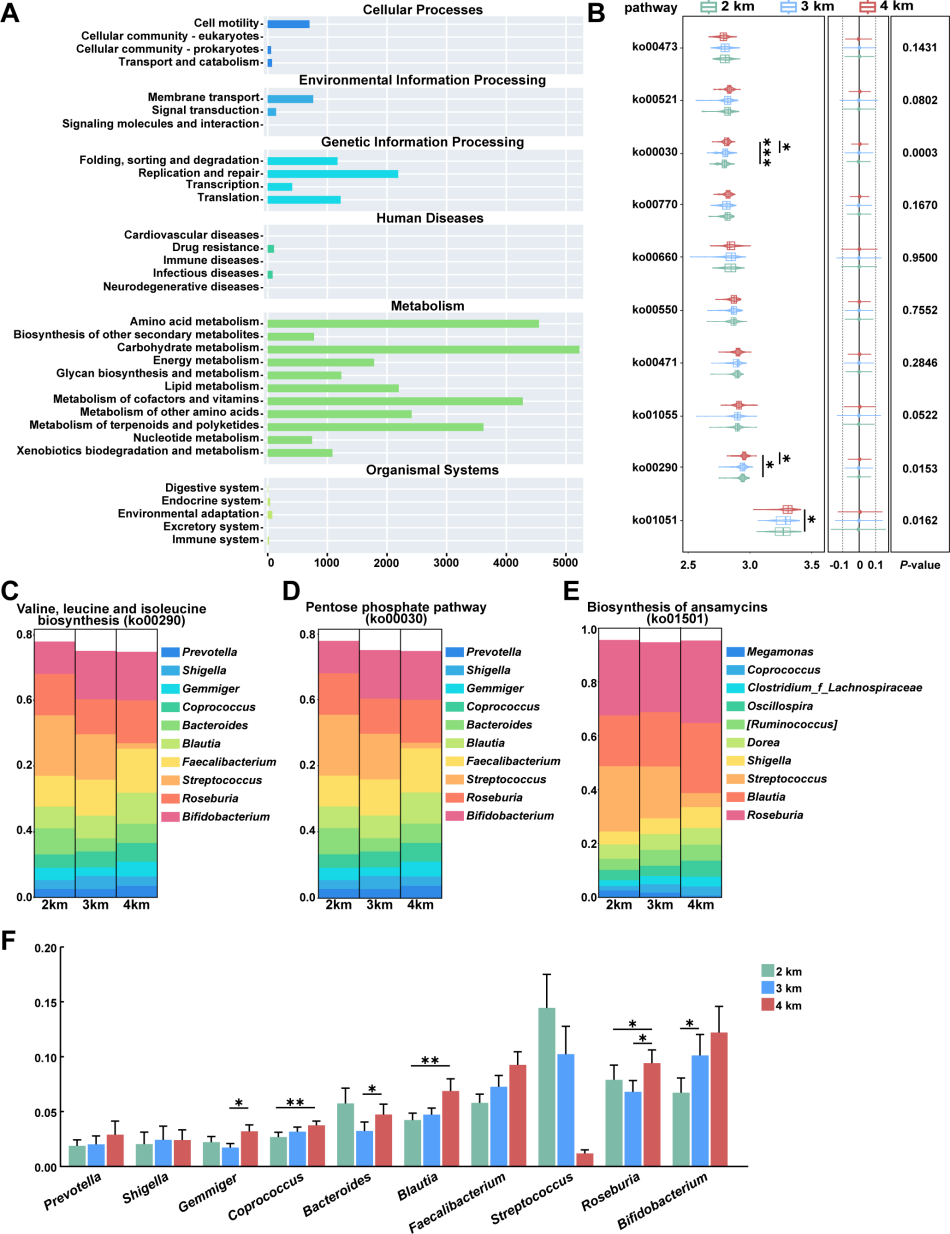
The functional modules of fecal microbiota exhibit distinct patterns across three study groups. (**A**) Enrichment profiles of functional modules. (**B**) Differential metabolic pathway analysis at the KO-level based on functional predictions. Statistical analyses were using the Kruskal-Wallis test with Dunn *post hoc* tests. (**C–E**) Relative contributions of microbial genera to the top 10 modules for ko01501, ko00030, and ko00290 pathways. (**F**) Top 10 genera contributing to the ko00030 and ko00290 pathway at the genus level. Data are presented as mean ± SEM. Statistical analyses were using the Kruskal-Wallis test with Bonferroni *post hoc* tests. *P < 0.05, **P < 0.01 and ***P < 0.001.

Predicted functional profiles derived from KEGG annotation revealed differences in microbial metabolic potential across altitude groups (Fig. 5A). At the pathway level, compared to the 2 km group, the 4 km group showed a significantly higher predicted gene abundance associated with the pentose phosphate pathway (*P* < 0.001), as well as with valine, leucine, and isoleucine biosynthesis (*P* = 0.031), and biosynthesis of ansamycins (*P* = 0.016; Fig. 5B). The microbial genera correlated with these functionally distinct intergroup abundance patterns. Relative abundances of the SCFA producers *Blautia* (*P* = 0.002), *Coprococcus* (*P* = 0.004), and *Roseburia* (*P* = 0.026) were significantly higher in the 4 km group than in the 2 km group. Furthermore, the 4 km group showed greater abundances of *Gemmiger* (*P* = 0.033) and *Bacteroides* (*P* = 0.041) compared to the 3 km group (Fig. 5C through F). The predicted functional profile pointed to enhanced energy and biosynthesis metabolism in the high-altitude microbiome, correlating with an increase in SCFA-producing bacteria.

### Associations between the EEG metrics and gut microbiota

**Fig 6:**
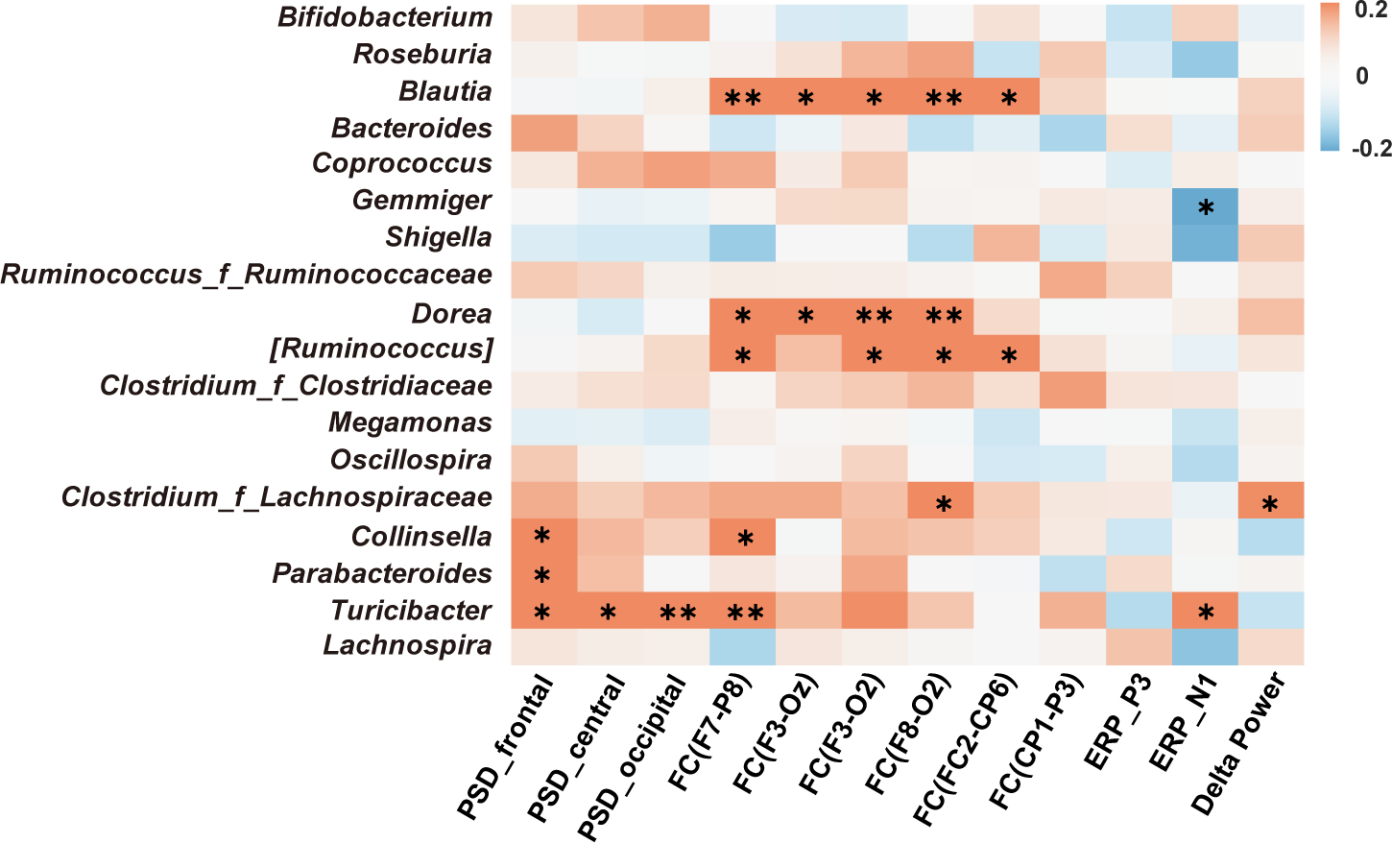
Significant correlations between differential abundances of gut microbiota and EEG indicators. The color bar indicates the Spearman correlation coefficients. *P < 0.05 and **P < 0.01.

The correlations between the LEfSe-identified abundant taxa and EEG metrics are summarized in Fig. 6. Resting-state delta PSD positively correlated with specific microbial genera, including *Collinsella* (*r* = 0.212, *P* = 0.029), *Parabacteroides* (*r* = 0.221, *P* = 0.022), and *Turicibacter* (*r* = 0.219–0.252, *P* < 0.05). Delta-band FC positively associated with the abundance of *Blautia* (*r* = 0.204–0.315, *P* < 0.05), *Dorea* (*r* = 0.204–0.283, *P* < 0.05), *Collinsella* (*r* = 0.232, *P* = 0.016), and *Turicibacter* (*r* = 0.254, *P* = 0.008). For task-state indices, a negative correlation was observed between Gemmiger abundance and N1 amplitude (*r* = −0.218, *P* = 0.024). In contrast, task-related delta power was positively correlated with the abundance of *Clostridium_f_Lachnospiraceae* (*r* = 0.197, *P* = 0.042). In addition, PSD was significantly positively correlated with several CVLT scores (*r* = 0.201–0.306, *P* < 0.05). FC was positively correlated with anxiety scores (SAS: *r* = 0.268–0.305, *P* < 0.05), sleep quality scores (PSQI: *r* = 0.221, *P* = 0.022) and CVLT scores (*r* = 0.215–0.234, *P* < 0.05; Fig. S3). The results indicated correlations between specific gut microbial taxa and EEG patterns, which were further associated with anxiety and sleep disturbances.

## DISCUSSION

In this study, we analyzed resting-state and task-state EEG to explore changes in brain function among indigenous residents at different altitudes. Furthermore, we investigated key alterations in gut microbial communities. The main findings are summarized as follows. (i) The 4 km group showed enhanced resting-state delta oscillations and frontal-occipital FC. During the oddball task, this group also exhibited elevated P3 amplitudes and increased parietal delta power compared to lower altitudes. (ii) Gut microbiota analysis revealed significant differences in composition, relative abundance (e.g., *Roseburia*, *Blautia* under *Firmicutes*) and metabolic pathways, with microbial changes in the 4 km group better reflecting adaptation to high altitude. (iii) The abundances of *Blautia* and *Dorea* were positively correlated with FC, and FC was further associated with higher anxiety and poorer sleep quality scores. This study provides a novel neuro-microbial perspective on high-altitude adaptation by integrating brain electrophysiology with gut microbiome data from indigenous populations.

### Resting-state delta enhancement and FC reorganization in high altitude

The brain is highly dependent on oxygen, and its normal function relies on a continuous and stable blood supply (33). During acute high-altitude exposure, cerebral blood flow is redistributed to prioritize brain regions associated with core cognitive functions such as perception, cognitive control, and problem-solving, while relatively reducing support for other cognitive abilities (34). These adaptive changes in cerebral hemodynamics may further influence patterns of neuroelectrical activity. Xie et al. reported that acute hypoxia at an altitude of 4 km induces increased delta oscillation power (35). Consistent with this, we observed elevated delta power across most brain regions with rising altitude. Delta oscillations play a key role in neural integration and the maintenance of homeostasis, particularly in attention regulation, salience detection, and subliminal perception (36, 37). Therefore, elevated delta likely represents a compensatory mechanism that preserves basic brain functions under oxygen deprivation. Notably, our study extends this observation to indigenous high-altitude populations. The frontal cortex is involved in cognitive and emotional regulation (38). Prolonged high-altitude exposure can lead to impairments in attention and inhibitory control (39, 40), possibly linked to prefrontal dysfunction. Enhanced FC between the prefrontal and occipital regions was observed in our study. This may reflect a compensatory reorganization that facilitates the integration of perceptual and executive processes under high altitude. Additionally, a significant decrease in the alpha band during acute high-altitude exposure has been reported (35, 41). The reduction in alpha activity reflects weakened FC within the central nervous system, which may lead to cognitive deficit (42). However, Zhao et al. (43) further observed that as individuals acclimatize to high-altitude conditions, alpha activity may return to normal or even increase. The absence of significant group differences in alpha oscillations in our study may also support the notion that long-term adaptation normalizes this frequency band. Collectively, the increase in delta power and enhanced connectivity in the occipital and frontal regions may illustrate an adaptive mode in high-altitude indigenous populations.

### Enhanced P3 and delta power during cognitive task in high altitude

In the ERP analysis, the 4 km group exhibited significantly larger P3 amplitudes over parietal regions compared to the other two groups. The P3 component is mainly elicited during executive control tasks (44) and is typically enhanced under conflict conditions (45), reflecting cognitive processes such as attention allocation, working memory, and information processing. Since P3 amplitude is thought to reflect the efficiency of resource allocation (46), this indicates that high-altitude residents may mobilize more attentional resources to resolve target-distractor conflict. Interestingly, the N1 component was significantly lower in the 4 km group. The N1 component is an early negative wave that occurs after stimulus onset and reflects initial sensory processing (47). A reduced N1 amplitude may indicate decreased resource investment during early perceptual stages (48). We therefore speculate that the relatively reduced N1 amplitude in residents at 4 km reflects improved efficiency in early perceptual processing. This enhanced efficiency may, in turn, support later P3-related cognitive operations, which require greater resource investment. Consistent with resting-state delta enhancement, elevated parietal delta power was found during the oddball task in the 4 km group. Delta activity is closely associated with target detection, and its augmentation during task engagement suggests enhanced efficiency in processing target stimuli (49, 50). This may also support adaptation in high-altitude populations.

### Gut microbiota restructuring in response to high altitude

Recent research underscores the importance of gut microbiota in host adaptation to high-altitude environments (51). After migration to high altitude, the gut microbiota gradually shifts toward a profile resembling that of indigenous populations. Furthermore, some of these altitude-associated microbial features can persist even after returning to lowland conditions (52). In line with this adaptive plasticity, our study found structural differences in the gut microbiota across altitude groups. We observed that several *Firmicutes* members were altitude sensitive*,* with *Roseburia*, *Blautia*, and *Coprococcus* significantly enriched at 4 km. This aligns with a meta-analysis that identifies *Roseburia*, *Faecalibacterium*, *Dorea*, and *Coprococcus* as common microbial biomarkers at high altitude (53). This helps explain the dominance of *Firmicutes* at high altitude and suggests their essential role in host adaptation to these conditions.

### Gut microbiota composition is associated with altered EEG activity at high altitude

Correlation analysis further revealed that the relative abundances of *Blautia* and *Dorea* were positively associated with delta band FC. *Blautia* and *Dorea* contribute to SCFA production (54). *Blautia producta* mediates immunomodulation through SCFA, as evidenced by the attenuation of neuroinflammation in Parkinson’s disease (55). In addition, SCFA also contribute to neural regulation. These accumulate in the hypothalamus to stimulate the synthesis of the inhibitory neurotransmitter gamma-aminobutyric acid (GABA) (56). GABA represents the primary inhibitory neurotransmitter in the mammalian central nervous system and acts in synergy with acetylcholine (57). This is likely to reinforce the function of key inhibitory networks within the thalamocortical system (58, 59), thereby promoting enhanced phase synchronization across brain regions in the delta frequency band. These findings collectively suggest that the gut microbiota and brain function are interlinked and may support the role of gut-brain interaction in high-altitude adaptation.

We further discovered that *Blautia* may also exert a potential influence on behavior in high-altitude populations by gut-brain interaction. Anxiety and sleep disturbances are common comorbidities among outpatients in high-altitude areas (60). This may be linked to hyperarousal caused by neurotransmitter system dysregulation, including dysregulation involving cholinergic and GABA mechanisms (61). This may help explain our finding that *Blautia* could affect behavior through GABA-related pathways. Studies have found that *Blautia* is related to anxiety-like behaviors in a mouse model of autism (62), indicating that *Blautia* may participate in anxiety regulation. However, direct evidence for this microbiota-brain-behavior crosstalk remains insufficient. Consequently, our future research will further investigate the underlying mechanisms of the microbiota-EEG-behavior interaction.

This study has several limitations that should be acknowledged. First, in addition to chronic hypoxia, other common physiological changes, such as oxygen saturation and hematocrit, may also shape the effects of high altitude on gut-brain interaction. Future studies that measure these factors systematically could help clarify how high altitude works as a whole. Second, although our findings pointed to distinct cognitive traits in high-altitude natives, their genetic basis was not determined. Genes linked to hypoxia adaptation, such as EPAS1, might play a role. Future work, including genetic data, could better elucidate how heredity influences these cognitive features. Third, even though we limited the study to one region, individual differences in body weight and diet could still affect gut bacteria. The inclusion of measures such as BMI and dietary records in future research would help to elucidate gut microbiota more precisely. Finally, although our findings provide preliminary evidence for mediated changes by gut microbiota and brain interaction, further causal validation is warranted.

### Conclusion

This study reveals that indigenous high-altitude populations exhibit distinct neuroadaptation and an enriched SCFA-producing gut microbiome. The relative abundance of these microbiota positively correlated with brain activity and could thereby further influence mood. These findings help us understand the role of the gut-brain interaction in the high-altitude adaptation.

## Data Availability

The data used in this work are available for academic use at the following link: https://pan.baidu.com/s/1owXs9OJn0P9hPpQsdCg2Vg?pwd=m447. Any further questions should be addressed to corresponding author Wen Wang.

## SUPPLEMENTAL MATERIAL

Supplemental figures and tables msystems.01692-25-s0001.pdf Fig. S1 to S3; Tables S1 and S2.

ASM does not own the copyrights to Supplemental Material that may be linked to, or accessed through, an article. The authors have granted ASM a non-exclusive, world-wide license to publish the Supplemental Material files. Please contact the corresponding author directly for reuse.
