# Supplementary material for: EEG and gut microbiota response patterns in high-altitude indigenous populations
Source: mSystems. 2026 Mar 4;11(3):e01692-25. doi: 10.1128/msystems.01692-25 (PMC13011389; doi:10.1128/msystems.01692-25)
Supplement: Supplemental figures and tables — Fig. S1 to S3; Tables S1 and S2. [file msystems.01692-25-s0001.pdf]

Supplementary figures

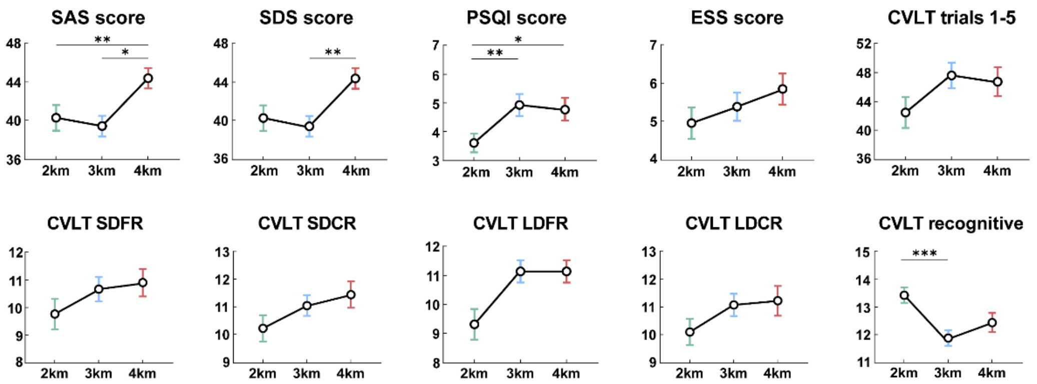

Figure S1. Statistics of differences in neuropsychological tests. Data were presented as mean ± SEM. Statistical analyses were analyzed using the Kruskal-Wallis test with Bonferroni *post-hoc* tests. *$P < 0.05$, **$P < 0.01$, ***$P < 0.001$. Abbreviations: SAS, Self-rating Anxiety Scale; SDS, Self-rating Depression Scale; PSQI, Pittsburgh Sleep Quality Index; ESS, Epworth Sleepiness Scale; CVLT, California Verbal Learning Test; CVLT SDFR, CVLT short delay free recall; CVLT SDCR, CVLT short delay cued recall; CVLT LDFR, CVLT long delay free recall; CVLT LDCR, CVLT long delay cued recall.

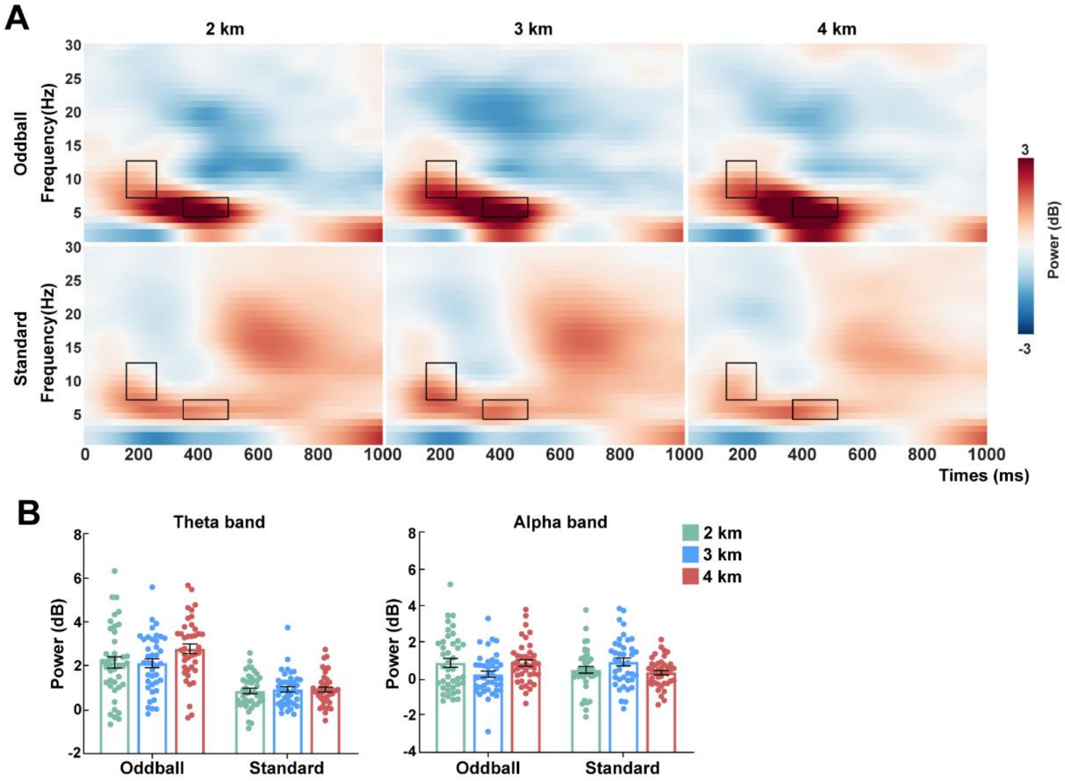

Figure S2. The EEG time-frequency characteristics of the oddball task. (A) Grand average time-frequency plots elicited by standard and oddball stimuli at parietal electrodes (P3, Pz, P4). (B) Topographic maps of the theta band (350-500 ms) and alpha band (150-250 ms). (C) Mean power of theta and alpha bands. Data were presented as mean ± SEM. Statistical analyses were performed using two-way ANOVA followed by Bonferroni *post-hoc* tests. Abbreviations: EEG, Electroencephalography.

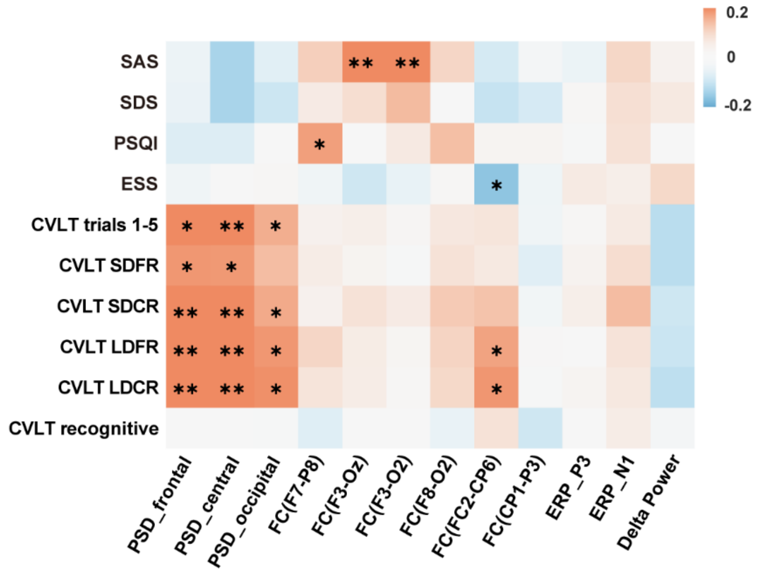

Figure S3. Significant correlations between neuropsychological tests score and EEG indicators. The color bar indicates the Spearman correlation coefficients. *P < 0.05, **P < 0.01. Abbreviations: SAS, Self-rating Anxiety Scale; SDS, Self-rating Depression Scale; PSQI, Pittsburgh Sleep Quality Index; ESS, Epworth Sleepiness Scale; CVLT, California Verbal Learning Test; CVLT SDFR, CVLT short delay free recall; CVLT SDCR, CVLT short delay cued recall; CVLT LDFR, CVLT long delay free recall; CVLT LDCR, CVLT long delay cued recall.

Supplementary table

Table S1 Statistical summary

Parameter	Statistical methods	*Post-hoc*
**Demographic data**		
Age, education	Kruskal-Wallis test	Bonferroni
Sex	Chi-square test	/
**Neuropsychological data**		
SAS, SDA, PSQI, ESS, and CVLT	Kruskal-Wallis test	Bonferroni
**EEG data**		
Behavioral performance (accuracy, RT)	Kruskal-Wallis test	Bonferroni
PSD	Kruskal-Wallis test	Bonferroni
FC features	T-test	FDR
ERP and time-frequency features	Mixed-design analysis of variance	Bonferroni
**Gut microbiota data**		
Alpha diversity, beta diversity, and relative abundance	Kruskal-Wallis test	Bonferroni and Dunn
Functional Differences analysis	Kruskal-Wallis test	Dunn
**Correlation**	Spearman	/

Abbreviations: SAS, Self-rating Anxiety Scale; SDS, Self-rating Depression Scale; PSQI, Pittsburgh Sleep Quality Index; ESS, Epworth Sleepiness Scale; CVLT, California Verbal Learning Test; EEG, electroencephalography; RT, reaction time; PSD, power spectral density; FC, functional connectivity; ERP, event-related potential.

Table S2 Behavioral performance of the experimental conditions related to the three groups

**Parameter**	**2 km (n = 41)**	**3 km (n = 40)**	**4 km (n = 40)**	***P*-value**
Oddball accuracy (%)	100.00 (3.84)	100.00 (3.31)	100.00 (4.98)	0.589
Standard accuracy (%)	100.00 (0.70)	100.00 (0.72)	100.00 (0.71)	0.934
Oddball RT (ms)	452.00 (71.17)	437.22 (87.78)	430.92 (52.42)	0.669

Data were presented by median (IQR).
